# Effects of Social Support Interventions on Medical Patient Survival: A Meta-Analysis of Non-Randomized Clinical Trials

**DOI:** 10.3390/healthcare14020277

**Published:** 2026-01-21

**Authors:** Ksenia Illinykh-Bair, Timothy B. Smith

**Affiliations:** Department of Counseling Psychology and Special Education, Brigham Young University, Provo, UT 84602, USA

**Keywords:** coping skills, emotional resilience, mortality, motivation, psychological distress, psychology, support groups, tertiary prevention

## Abstract

**Background**: Prior research confirms that social support promotes resilience among medical patients with chronic illness. Beyond emotional benefits, research has increasingly shown the importance of social support on physical health outcomes. Therefore, identifying and evaluating interventions that increase social support among medical patients with chronic conditions is a priority for healthcare. **Methods**: This meta-analysis summarized data from 39,493 medical patients across 14 non-randomized trials that had been identified by a prior review of the survival benefits of social support interventions. **Results**: Across four studies reporting hazard ratio data, the results failed to reach statistical significance (HR = 2.10, 95% CI = 0.99 to 4.48, *p* = 0.0546), and the results of ten studies reporting odds ratio data were of smaller magnitude (OR = 1.27, 95% CI [0.72, 2.23], *p* > 0.05). Heterogeneity characterized both the odds ratio data (I^2^ = 53%; *Q* = 18.1, *p* = 0.03) and hazard ratio data (I^2^ = 89%, *Q* = 23, *p* < 0.001). A notable finding was that studies with longer periods of data collection showed longer survival among medical patients receiving social support. **Conclusions**: Long-term observations may be necessary for the survival benefits of social support interventions to become apparent. Further research with a larger pool of data from long-term follow-up studies will be needed to establish firm conclusions.

## 1. Introduction

Social support benefits multiple aspects of human functioning [1,2,3]. Social support enhances networks of practical assistance, mutual reliance, belonging, information, and guidance [4], all promoting well-being. Through social support, individuals develop greater self-esteem and sense of control [5], and better cope with adversity [6]. Moreover, social support promotes physical health [7,8,9].

A recent major scientific report emphasized the importance of social support on physical health [10]. Social support particularly benefits people experiencing difficult circumstances, such as physical illness and corresponding distress. Therefore, finding ways to increase social support among medical patients is a priority for future healthcare research.

Directly addressing that need, this meta-analysis evaluates the effectiveness of social support interventions in healthcare settings that are intended to improve the physical condition of medical patients. Social support interventions can include emotional, informational, and instrumental components [11], each contributing to a patient’s recovery. Emotional support—offered by caregivers, family, or peer groups—provides comfort, empathy, and reassurance, buffering the emotional toll of illness [12,13]. Informational support includes the provision of knowledge and guidance by healthcare professionals, helping patients make informed decisions about their treatment and care [12,14]. Instrumental support involves tangible assistance, such as home care services or logistical help, ensuring that patients have the resources they need to maintain their quality of life [15]. The comprehensive benefits of social support interventions deserve evaluation in healthcare settings.

To meet a notable gap in the literature, this meta-analysis specifically examines the effectiveness of social support interventions evaluated through non-randomized controlled trials (NRCTs). While randomized controlled trials (RCTs) are frequently recognized as the most reliable method in evidence-based research, they are not always feasible or ethical in the context of social support studies [16,17]. Specifically, medical patients experiencing life-threatening conditions are much more likely to seek out the psychosocial support that they need than to agree to receive no treatment by participating in a control group. Thus, RCTs may completely misrepresent the experiences of medical patients needing emotional support. While RCTs better control for threats to experimental internal validity, NRCTs demonstrate real-world applicability of interventions, tend to have larger numbers of participants, and address the ethical and practical challenges often associated with RCTs [18,19,20]. Moreover, although a medical patient experiencing minimal distress may be willing at a start of a research study to agree to be assigned to a control group, when their medical condition worsens or distress increases, they are likely to find other means of support rather than remain without a support intervention in the face of a life-threatening illness. Such undisclosed patient cross-over to another intervention can invalidate an RCT control group. By focusing on NRCTs with self-selected treatment conditions, this meta-analysis evaluates the effectiveness and practical applicability of social support interventions on the survival of medical patients actively seeking support, differentiated from those who do not. The benefits of this evaluation are many, since psychosocial interventions ostensibly could be more effective for those actively seeking them.

This meta-analysis also aims to fill a second gap in the literature by identifying patient-specific and intervention-specific factors that may influence their effectiveness. Few meta-analyses of patient survival in NRCTs have synthesized the effects of such variables as treatment duration and length of follow-up across patients with different medical conditions, for instance. By synthesizing the outcomes of treatments provided in naturalistic settings and then evaluating how the treatment differences influence patient survival, this meta-analysis strives to offer useful information about the effectiveness of social support interventions in medical settings. It seeks to identify patterns and trends that can inform future research, improve intervention design, guide clinical practices, and provide actionable insights for healthcare providers.

### 1.1. Literature Review

Prior research has identified many causal pathways through which social support improves physical health. Behavioral and emotional pathways include: (a) improved health behavior and habits [3], such as dietary choices and increased physical activity [21], with family support being particularly influential [22,23,24,25]; (b) stress management support [13]; (c) improved knowledge about treatment or lifestyle options, which improves medical patient sense of control and trust [26,27]; (d) engagement in meaningful activities [28], and fostering a sense of purpose [29]; (e) improved positive self-concept and ability to face medical conditions [30]; (f) rebounding from setbacks and emotional resilience [31,32]; (g) adaptive coping strategies such as emotional regulation, problem-solving, and time-management [33,34]; (h) compliance with medical advice [35,36]; and (i) stronger ability to perform daily tasks and self-care [37].

Beyond behavioral and emotional influences, physiological pathways explaining the benefits of social support on physical health include: (a) strengthened immune function [38,39,40]; (b) lower heart rate, cortisol, and blood pressure reduce the allostatic load that reflects continuous wear and tear on the body [40,41,42]; (c) diminished pain perceptions [43], and (d) improved neuroendocrine responses, oxytocin levels [44], and reduced cortisol levels [45].

In particular, medical patients have a strong need for social support [46,47]. In healthcare settings, physicians, nurses, social workers, and chaplains play a vital role in supporting medical patients, especially those with chronic diseases [11]. Emotional empathy and reassurance provided by medical teams helps patients reduce anxiety, helplessness, and depression [48,49,50], and improve their perceptions of treatment and disease progression [51,52].

Healthcare professionals can provide a wide variety of social supports that facilitate patients’ management of chronic health conditions. Research shows that psychosocial behavioral support interventions [53], health information supports [54], home-based care services [55], and nurse phone contact [56], all improve patient outcomes, reduce hospitalizations, and enhance overall quality of life.

Social support is crucial at any time during the clinical course of diagnosis, treatment, and rehabilitation. When patients are first diagnosed and begin treatment, they often feel weak and vulnerable, oftentimes exacerbated by altered sleep patterns, compromised nutritional intake, fatigue, and pain [39]. During treatment of chronic illnesses, the persistent pain or discomfort can cause fatigue and diminished morale. After treatment and during recovery, patients are susceptible to a condition referred to as “posthospital syndrome” [57]. Research has shown that a lack of posthospital social support services diminishes patients’ ability to recover at home successfully, which ultimately decreases survival [39,58].

Psychosocial support interventions can include group, individual, and home-based services. For instance, support groups help medical patients to find ways to conserve energy, set realistic goals, and manage their rest and activities more effectively [59]. As a specific example of a support group [60], observed patients with coronary heart disease (CHD) over one year and found lower LDL cholesterol level, less weight gain, improved relaxation behaviors, and a trend toward adherence to medication. Overall, many research studies of support groups show that group interpersonal connections benefit patients with chronic conditions to improve their self-esteem, emotional resilience, self-acceptance, and coping [61,62,63,64,65,66].

Beyond medical patient support groups, another form of healthcare delivery of social support involves home visits by medical team members trained to provide emotional support or counseling, while also performing evaluations and administering medication [67]. Home-based social support delivery has been found to reduce hospital readmissions as well as patient depression and anxiety [55]. Home-based services are also less expensive than inpatient care [68,69], and relieve the travel burden for patients in outpatient care [70]. An early study found survival rates of 67% in the intervention group compared to 40% in the control group [71]. Other benefits of home-based supportive services for patients and their families include a sense of normalcy, strengthened physician–patient relationship, and improved patient-centered care [70].

### 1.2. Statement of Purpose

According to prior research, there is a pressing need for increased social support among medical patients [72]. There is also a need to review existing research to provide clarity about the effectiveness of social support interventions in improving survival among medical patients. Building on a prior review of randomized experiments [53], this meta-analysis evaluates data on the survival benefits of social support interventions among medical patients by restricting results to an evaluation of interventions conducted without participant randomization to improve understanding of outcomes in more naturalistic settings.

### 1.3. Rationale for Evaluation of Non-Randomized Controlled Trials

Non-randomized controlled trials can provide meaningful scientific information on the applicability of research across various populations, complementing results that may differ from randomized controlled trials [73]. A primary consideration involves practical and ethical constraints [74,75,76], such as when patients are physically ill, allowing them to self-select the type of support that they prefer.

A second consideration involves generalizability and real-world contexts. While RCTs usually involve highly controlled settings, NRCTs typically occur in pre-existing settings, allowing for an evaluation of how interventions may function outside of controlled conditions. Moreover, participants recruited from naturally occurring settings may be more representative of individuals in those settings and may also have more diverse characteristics than individuals willing to volunteer for an RCT. Studies involving social support interventions in fields such as healthcare often occur in naturalistic settings [76], so it is important to evaluate those studies and compare their results to RCTs rather than omit them.

A third consideration involves feasibility and sample size. Since RCTs involve greater complexity and typically require additional resources to complete, collecting data with large samples in RCTs can prove challenging. Non-randomized studies typically cost less, and this feasibility can result in a larger number of participants as a trade-off [73,76,77].

A fourth consideration involves potential control group members’ non-disclosure of parallel interventions or support-seeking. While participants in RCTs have no choice about the conditions received in the research study, participants in non-randomized experiments typically had selected the type of condition they experienced based on preference [74,76]. Individuals who intentionally selected social support or not would logically be more satisfied with their choice than someone assigned to conditions by a researcher, such that those who had hoped to receive social support but were assigned to a control condition may go ahead and seek out alternative forms of social support, thus confounding the results of an RCT. While randomization of the RCTs minimizes many other forms of research bias, the design creates complexities when participants receive social support from a source outside the RCT. Those who seek social support outside the RCT may also be less willing to divulge that information when asked by researchers, thus problematizing the nature of the control group, even when participant cross-over is tracked by researchers.

Given these several considerations, rather than limiting data sources to RCTs, there are multiple benefits of comparing results across research designs, RCTs, and NRCTs. When NRCTs yield similar results to RCTs, that provides the field with data triangulation [78,79]. When the results diverge [80], that can inform future evaluations of plausible contextual factors influencing the findings across studies. Thus, data comparisons produce a deeper understanding of the intervention’s effects across different contexts [74,81,82]. Such comparisons of results across multiple studies align with the strengths of meta-analyses [82,83].

### 1.4. Research Questions

This meta-analysis addressed two research questions:

1. What is the overall random-effects weighted effect size of social support interventions on medical patient survival in non-randomized trials?
2. What study, patient, and intervention characteristics produce differences in results across studies that influenced the effect of social support interventions on medical patient survival?

## 2. Methods

We conducted a meta-analysis of previously identified research publications. In an extension of a prior meta-analysis specific to RCTs [53], we located and analyzed the 14 NRCTs not analyzed in that prior meta-analysis. In that article entitled Effects of Psychosocial Support Interventions on Survival in Inpatient and Outpatient Healthcare Settings: A Meta-Analysis of 106 Randomized Controlled Trials, Smith and colleagues had followed PRISMA guidelines and searched for both published and unpublished RCTs that examined the impact of psychosocial support interventions on the survival of medical patients. Their search from January 1980 to October 2020 used these databases: Embase, Medline, Cochrane Library, Alt Health Watch, CINAHL, PsycINFO, Social Work Abstracts, and Google Scholar. In this project, we evaluated the NRCTs found in those searches without conducting additional searches, thus missing eligible studies. The rationale for this approach is that to our knowledge, no prior review specific to NRCT data had been conducted, so we sought the benefits of a rapid review without an exhaustive literature search [84]. When research teams disband or lack resources, publishing available data remains beneficial to the field and reduces publication bias [85,86]. Moreover, this approach was justified by the fact that in a prior review “abbreviated literature searches often led to identical or very similar effect estimates as comprehensive searches, with slightly increased confidence intervals” ([87], p2). Thus, delimited meta-analyses can guide future research and, in this instance, also establish initial findings to guide triaging of patients with chronic health conditions requiring psychosocial support. While this project is not a systematic review and involves a narrow sample of articles possibly available, it is a meta-analysis of previously unreported data that can inform healthcare research and practice regarding medical patient self-selected support interventions.

### 2.1. Study Selection Inclusion/Exclusion Criteria

This meta-analysis analyzed previously identified NRCTs that reported medical patient survival outcomes following real-time interventions offering psychological, emotional, and/or social support. The studies evaluated patients with life-threatening health conditions who were recruited from curative/rehabilitative healthcare settings, hospitals and outpatient clinics. The following Population, Intervention, Comparison, and Outcome (PICO) criteria were applied to published or unpublished reports in any language.

#### 2.1.1. Participants/Population

We included studies of medical patients in curative or rehabilitative care for a disease that would result in death if untreated. Patients who only had dementia or mental illness without a physical health condition were excluded because those conditions would interfere with the effectiveness of psychosocial interventions. Additionally, individuals recruited from non-health care settings, such as Alcoholics Anonymous or other 12-step support groups, were not included. For studies with overlapping participants across multiple publications, we chose data from the longest follow-up period or, if follow-up durations were the same, the largest sample size.

#### 2.1.2. Interventions

This meta-analysis evaluated psychosocial interventions. Eligible interventions provided real-time social, emotional, and/or psychological support, either in-person or through technology facilitating remote connections (e.g., telephone, computer, tablet). These interventions could be delivered individually or in group settings (the authors coded for intervention format). Given that most psychosocial support interventions in the literature incorporate multiple elements, the study included interventions with mixed components (e.g., group psychotherapy, nurse visits, and telephone support) and coded for differences to compare outcomes. Interventions that only provided psychoeducation or disease management, as well as those limited to one-on-one psychotherapy (which is considered a distinct intervention type), were excluded. We also excluded interventions specific to health behaviors and substance abuse, since those involve additional components. Since the focus of this meta-analysis was on curative and rehabilitative interventions’ impact on longevity, the authors excluded interventions that did not focus on prolonging life, such as hospice and palliative care. Additionally, interventions involving nonhuman support (e.g., spiritual practices, pets) were not included.

#### 2.1.3. Comparator(s)/Control

This meta-analysis focused solely on non-randomized controlled trials. The authors included studies in which the comparison group either did not receive any equivalent psychosocial intervention (e.g., medical treatment as usual) or received non-supportive attention, such as psychoeducational information about disease progress and disease management (and the authors coded the differences). The authors excluded studies that only compared one bona fide psychosocial support intervention to another.

#### 2.1.4. Outcome

We included articles in which medical patient mortality was confirmed by objective sources. We evaluated data on medical patient survival in terms of both: (a) survival time (reported as hazard ratios), and (b) binary survival at a fixed time point (reported or calculated as odds ratios). Data that combined mortality with morbidity/re-hospitalization were excluded.

### 2.2. Data Extraction

In the authors’ prospective planning, they aimed to minimize the chances of human error in data coding by having each article coded by a pair of raters. Additionally, a separate pair of raters independently coded the same article. This redundancy was designed to enhance the accuracy of both the coding process and data entry. When discrepancies occurred in the initial review of manuscripts regarding their inclusion, the team met to review those studies. Inclusion research exclusion was based on consensus after reviewing the wording of the manuscript and the study criteria.

When a study reported multiple effect sizes at the same point in time (e.g., from different subsamples), the authors averaged the values, weighted by standard error (SE), to avoid violating the assumption of independent samples. For studies with effect sizes across different time points, the authors used data from the longest follow-up period (or the largest sample size if the follow-up durations were the same).

The authors prioritized odds ratio (OR) and hazard ratio (HR) data as effect sizes, but if other statistics were provided (e.g., regression coefficients, Cohen’s d, frequency counts), they converted these to ORs using the validated Campbell Collaboration’s effect size calculator [88]. While multivariable models were preferred, the authors coded univariate data when necessary. In cases where mortality was monitored but no deaths occurred in either condition, the authors recorded the effect size as OR = 1.

In this meta-analysis, we evaluated data in terms of patient survival. Therefore, for both OR and HR data, values above 1 indicated improved patient survival in the intervention group, while values below 1 indicated that patients in the control group experienced better survival than those receiving the intervention.

Information from studies was reported in a spreadsheet. That information included different aspects of the study, listed below.

- Information about the study: research design, type of control condition, year of study initiation, name of country;
- Participant characteristics of age, sex, medical diagnosis at intake, percentage of patients dying by endpoint, healthcare setting (inpatient, outpatient, or both);
- Information about the intervention type (e.g., one-on-one support, support group), focus (e.g., behavioral support, social/emotional support), delivery (e.g., in-person, telephone/online), number of sessions, length of sessions, duration of sessions in months, and length of follow-up period after completion of intervention;
- Effect sizes in terms of survival time (reported as hazard ratios) or binary survival at a fixed point in time (reported/calculated as odds ratios) and corresponding standard error.

Extracted data were cross-checked to identify discrepancies. Discrepancies were resolved through discussion following scrutiny of the manuscript to the point of consensus or arbitration by the last author.

### 2.3. Data Analyses

Our team analyzed omnibus effect sizes separately for OR and HR metrics. This approach also cleanly separated the unadjusted results from the adjusted results: all OR values were calculated from raw frequency data and all HR values were calculated from multivariate models. To observe effect size heterogeneity, we computed both *Q* and I^2^. Following those analyses, we evaluated study, intervention, and participant characteristics in a series of subgroup analyses and meta-regressions. The possibility of publication bias was evaluated using the methods of Egger’s and Peter’s regression tests, funnel plots, and the trim and fill method. All analyses were conducted using random-effects weighted models calculated by STATA 18.

## 3. Results

### 3.1. Study Characteristics

Non-redundant effect sizes were extracted from 14 NRCTs. Data involved a total of 39,493 participants, whose average age was 51.6 years (SD = 11.5, range = 34.7 to 67.1), with an average of 58% females (SD = 29.7). Seven studies were conducted in the United States, three in Europe, three in Africa, and one in Australia. Eleven studies evaluated patients receiving outpatient treatment, one involved hospitalized inpatients, and two studies involved patients receiving either inpatient or outpatient treatment. Four studies involved patients with cardiovascular disease (CVD), five studies had patients with cancer, four patients had HIV/AIDS, and one had patients with kidney disease. Eleven of the studies were conducted in a healthcare office or clinic, one study was conducted through telephone/video interaction, and two studies were conducted through a combination of formats.

Participants received the following types of interventions: four studies involved individual one-on-one support sessions; five involved group-based interventions; and five included a combination of individual and group sessions, with a spouse or caregiver invited to attend the sessions. Interventions were conducted by peers with similar conditions in two studies, by non-professionals or volunteers in two studies, by nurses, medical assistants, or clinic/hospital “staff” in four studies, and by social workers or counselors or mental health professionals in six studies. On average, each intervention session lasted 84.5 min (SD = 36.4, range = 20–150) with 21.9 total sessions (SD = 32.3, range = 3–120) over 9.6 months (SD = 15.1, range = 1–54). The length of time from the start of the intervention to final data collection averaged 31.4 months (SD = 32.6).

Across the included studies, an average of 16.6% of participants were deceased at the time the effect size was reported (SD = 20.8). Reported causes of mortality included all-cause mortality in eleven studies, cardiovascular disease (CVD) in one study, cancer in one study, and AIDS-related mortality in one study. Descriptive information is provided in Table 1.

### 3.2. Odds Ratios of Patient Survival

A random-effects meta-analysis of 10 studies reporting unadjusted odds ratios demonstrated a non-significant pooled effect (OR = 1.27, 95% CI [0.72, 2.23]; z = 0.83, *p* = 0.41). These results indicate that the social support interventions did not significantly influence medical patient survival.

**Figure 1:**
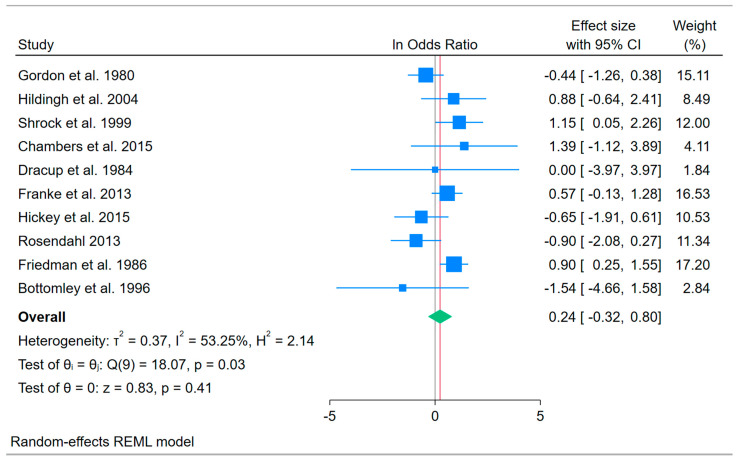
Forest Plot of Natural Log Odds Ratios for Medical Patient Survival Outcomes Across 10 NRCT Studies. Note: The vertical black line indicates “no effect” and the red vertical line indicates the observed weighted average effect [89,90,91,93,94,96,97,98,100,101].

Moderate heterogeneity characterized the findings across studies (τ^2^ = 0.37; I^2^ = 53.3%; H^2^ = 2.14; *Q*(9) = 18.1, *p* = 0.03). Figure 1 visually depicts study variation, which was not excessive. However, three studies in which almost all patients survived had wider confidence intervals, reflecting lower precision in the effect size due to limited mortality in both the intervention and control groups.

#### 3.2.1. Publication Bias Estimation with Odds Ratio Data

The results from the 10 studies were characterized by fairly symmetrical data points, and a trim-and-fill analysis identified no missing studies in the distribution. Furthermore, tests for small-study effects showed no evidence of publication bias: both Egger’s test and Begg’s test were non-significant (*p* > 0.10). Thus, publication bias did not appear to adversely influence the aggregate results of studies reporting odds ratios.

#### 3.2.2. Meta-Regression of Odds Ratio Data

To assess whether variability in patient survival could be attributed to differences in study characteristics, types of intervention, or patents’ characteristics, meta-regressions were conducted using odds ratio data (*k* = 10). Results across the 10 studies indicated that longer follow-up periods were significantly associated with increased likelihood of patient survival (*R*^2^ = 0.54, β = 0.02, SE = 0.01, *z* = 2.01, *p* = 0.04). These findings indicate that extended follow-up periods tend to detect an association between social support interventions and patient survival, suggesting that longer periods of data collection might be necessary to observe the benefits of the interventions. In support of this interpretation, the percentage of participants who had died at the time of data evaluation also demonstrated a positive relationship with the effect sizes, although it did not reach statistical significance (*p* = 0.07).

No other analyses involving study, patient, or intervention characteristics approached statistical significance across studies. Only the duration of data follow-up was associated with effect size variability.

### 3.3. Hazard Ratios of Patient Survival

Separate from the odds ratio data, a random-effects weighted analysis of the four studies reporting multivariate adjusted models yielding hazard ratios (HR) was conducted to understand the effects of social supports on medical patient survival. The results did not reach statistical significance (HR = 2.10, 95% CI = 0.99 to 4.48; *z* = 1.92, *p* = 0.0546), indicating similar outcomes of social support interventions compared to control conditions.

**Figure 2:**
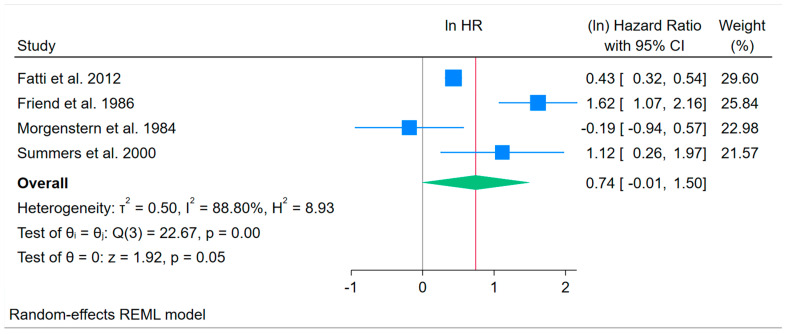
Forest Plot of Natural Log Hazard Ratios for Medical Patient Survival Outcomes Across 4 NRCT Studies. Note: The vertical black line indicates “no effect” and the red vertical line indicates the observed weighted average effect [92,95,99,102].

As shown in Figure 2, HRs varied widely, with one study demonstrating a non-significant adverse effect, one study reporting a mild effect, one study reporting a moderate effect, and a final study showing a large effect of the intervention on medical patient survival over time. We therefore conducted a leave-one-out analysis to re-estimate the pooled effects and improve interpretability of the results. In that analysis, three estimates remained non-significant, but omitting the lowest effect size yielded a statistically significant outcome for the remaining three studies (HR = 2.73, 95% CI = 1.30 to 5.78, *p* = 0.008). Omitting the middle two HR values resulted in only small differences from the previously reported omnibus values, but omitting the highest effect size yielded a much lower pooled estimate (HR = 1.53, 95% CI = 0.86 to 2.72, *p* = 0.145). Thus, the highest and the lowest of the four studies clearly influenced the pooled results reported earlier, in opposite directions. Overall, the HR results demonstrated significant heterogeneity (τ^2^ = 0.50; I^2^ = 88.80%; H^2^ = 8.93; *Q*(3) = 22.67, *p* < 0.001). Such a high level of heterogeneity suggested the need to assess study characteristics further to understand variability.

#### 3.3.1. Publication Bias Estimation with Hazard Ratio Data

Publication bias was not supported by either Egger’s or Begg’s tests for small-study effects, both of which were non-significant (*p* > 0.10). A funnel plot of four studies was too sparse to interpret with confidence. According to the trim-and-fill analysis, publication bias was unlikely, since no missing studies were implied, and the pooled effect size remained the same as the original values reported.

#### 3.3.2. Meta-Regression of Hazard Ratio Data

A random-effects meta-regression evaluated whether follow-up duration contributed to variability in hazard ratios. Longer follow-up periods were significantly associated with increased hazard ratios (*R*^2^ = 0.656, β = 0.012, SE = 0.005, *z* = 2.17, *p* = 0.03). Studies following up patients for long durations tended to report improved patient survival as a result of a social support intervention. Thus, the results of the HR data were similar to the results of the OR data, also indicating that patient follow-up duration explained effect size variability. No other study, participant, or intervention characteristic was associated with hazard ratio data in meta-regressions or sub-group analyses.

### 3.4. Evaluation of Risk of Bias

The conclusions of any meta-analysis depend on the quality of the studies evaluated. We therefore assessed risk of bias across all 14 studies using the 2025 Risk of Bias in Non-randomized Studies—of Interventions, Version 2 (ROBINS-I V2) [103]. We rated each study across all domains, with risk-of-bias judgments reported in Table 2. Overall, the studies were lacking in rigor. The unadjusted raw data from which odds ratio data were calculated did not control for important confounding factors, such as patient age and health status at intake. Some studies lost participants, with missing data possibly including mortality events. The notable methodological strength across studies was the reliability of the outcome variable, survival, an event confirmed objectively and unlikely to be influenced by research bias. Relatedly, given the single outcome (survival), researchers did not highlight certain findings across multiple measures. Across most studies, intervention strategies remained distinguishable from comparator strategies. Nevertheless, future research will need to take steps to mitigate risk of research bias.

## 4. Discussion

This meta-analysis investigated the relationship between social support interventions and survival among medical patients by synthesizing 14 non-randomized controlled trials identified but not analyzed in a prior meta-analysis of RCTs [53]. Across this limited number of studies, the survival data did not reach statistical significance but were of similar magnitude to findings in prior meta-analytic reviews.

### 4.1. Comparison of Present and Prior Findings

Prior research had suggested that social support interventions for medical patients are important for their well-being and could possibly influence their longevity [9,11,104]. Although previous results have varied, when medical patients receive psychosocial support [53], health education [54], in-home care programs [55,105], and nurse-led phone follow-ups [56], they have tended to experience better health outcomes and fewer hospital readmissions. An important contribution of the current meta-analysis was to examine the effectiveness of social support interventions in improving physical health in naturalistic settings, with patients selecting whether to participate in the social support intervention or not.

In the present meta-analysis in which the results did not reach statistical significance, the direction of both odds ratio and hazard ratio data indicated a beneficial effect of the intervention on medical patient survival (OR = 1.27 and HR = 2.10). In fact, the magnitude of the observed odds ratios was similar to the value of OR = 1.20 observed in a prior meta-analysis of 87 RCTs [53]. The similarity of the magnitude of the odds ratio results across the present and the prior meta-analysis may suggest independent replication. While the results of the current meta-analysis were slightly larger than the prior meta-analysis, they likely did not reach statistical significance due to the smaller number of studies reviewed, with associated lower statistical power.

It is also notable that the present meta-analysis of NRCTs and the prior meta-analysis of RCTs yielded similar results despite having evaluated different research designs [53]. Prior literature indicated that interventions evaluated through the RCTs may overstate intervention effects [106] due to the restricted and artificial nature of experimental trials. Specifically, RCTs might inadequately represent clinical settings and patient diversity, since not every patient is willing to sign up for a study in which they may not receive treatment. In contrast, NRCTs are more naturalistic and typically include broader participant samples [74,76].

Since non-randomized studies require fewer resources and expenses than randomized designs, they often include a larger number of participants as a trade-off [73,76,77]. NRCTs allow the participants to choose the condition they prefer in the study, which is more naturalistic, since participants who do not prefer to engage in social support initiatives would be unlikely to join them in real-world settings. Although NRCTs are susceptible to selection bias, they can provide findings that are more realistic than RCTs, with possibly broader generalizability of the research findings in community health settings [76].

This suggests that future research can continue to consider data from NRCTs, which are often more practical to conduct with existing programs, whereas RCTs require additional steps and resources. Moreover, since they are conducted in naturalistic settings, NRCTs may be more generalizable when evaluating long-term outcomes than randomized experiments in which participants may not receive their preferred treatments.

### 4.2. Contribution of This Meta-Analysis: Outcome Varied by Length of Patient Follow-Up

Meta-regression analyses were conducted to examine whether study-specific features (e.g., study-level, patient, or intervention characteristics) explained variability in patient survival. Longer follow-up data collection periods were associated with larger odds ratios (*p* = 0.04) and larger hazard ratios (*p* = 0.03). This remarkably consistent result across two different types of data demonstrates that the longer the researchers tracked the outcomes of medical patients, the more evident the effects of social support interventions on patient survival. The impact of social support interventions became more apparent over time, as more people approached mortality.

Across the studies included in this meta-analysis, the medical patient participants experienced an average likelihood of mortality of approximately 17% by the end of data collection. Although low death rates are optimal for the patients, relatively low incidence rates increase the width of confidence intervals and decrease the reliability of the results. That is, the effectiveness of any intervention remains difficult to observe when most patients survive irrespective of experimental condition. Most studies in this meta-analysis tracked patient survival for less than one year, yet it would have been preferable to track survival for longer periods of time.

The finding that the length of study follow-up was related to stronger intervention effects may also be explained by the nature of how diseases progress over time. For instance, patients may benefit most from social support interventions when they still retain sufficient physical vitality to actively engage in social settings. Previous research has suggested that social support is more imperative at the time of acute stress and crisis to help patients adapt to and cope with the diagnosis [107]. Literature also suggests that the continuity of social support is important across the stages of a patients’ medical journey from diagnosis to active treatment and recovery, with the most effective interventions occurring over an extended period, rather than simply a few sessions following initial diagnosis [39]. Boosting social support early and across the disease trajectory may prove more beneficial than when patients have only a few months to live or when they receive small intervention doses. Thus, future research needs to specifically examine how the length of follow-up periods overlaps with the stage of patients’ illness.

### 4.3. Limitations

The main limitation of the current meta-analysis is the small number of included studies (k = 14), resulting in a limited capacity to generalize the findings and draw reliable conclusions. Such a small sample of studies hinders the generalization of the findings to real-world settings. It is likely that more articles on the topic exist but were not identified in this project, which was specific to articles identified but not included in a prior meta-analysis of RCTs [53] to benefit from existing data in a review [84]. An exhaustive search for NRCTs will be needed in the future to confirm the effectiveness of social support interventions for medical patients in the NRCT settings.

Additionally, large heterogeneity was detected across the studies, especially in the hazard ratio data (I^2^ = 89%). This variability in findings may be a function of differences across studies in intervention types, study designs, patient populations, and follow-up durations. The differences in study findings make it difficult to conclude that the reported average effect sizes accurately represent the outcomes of social support interventions. Moreover, large variability with such low numbers of studies hinders the ability to identify differences in findings across study characteristics, since statistical power was low in this meta-analysis.

Research studies also varied in terms of procedures to reduce risk of research bias. The observed outcome of survival has low likelihood for research bias, but studies infrequently controlled for important confounding variables relevant to survival. Some studies lost participants during the intervention phase and follow-up, and many did not report procedures. This greatly reduces confidence in study outcomes.

Finally, a limitation of the studies included in this meta-analysis is that although they reported objective outcome data, they provide no specific evidence regarding causal factors. Social support interventions impact many variables, including increased medical treatment utilization and treatment plan adherence [108,109,110], reduced emotional distress [111], and increased self-control, self-awareness, self-esteem, emotional resilience, self-acceptance, and coping [61,62,63,64,65,66,112]. Each of these factors may contribute to long-term health benefits, but we do not yet know which are most influential in improving rates of medical patient survival. Future research needs to specifically measure and compare different causal pathways, such as contrasting intrapersonal emotional resilience with the pragmatic benefits of social capital, such as improved caregiver coordination and attentiveness, social modeling, and health information provided by peers with the same medical condition, and access to additional methods for healing recommended/provided in broader social circles.

### 4.4. Recommendations for Future Research

Overall, the quality of research evaluating psychosocial support interventions must improve. Researchers need to measure important potential confounds, such as patient age and initial mental and physical health status, and then control for those variables in statistical models. They need to explicitly track patient cross-over between control and intervention conditions, since deteriorating patients may stop attending support interventions or may seek them out if they have not been receiving them. They need to design their studies to align with the ROBINS-I domains [103] and then report their procedures clearly.

As research accumulates, future reviews of the effectiveness of patient-preferred social support interventions evaluated by NRCTs will be needed. Since individual patients have unique situations and characteristics, they may prefer certain social support interventions over others. For example, it might be more beneficial for some medical patients to receive one-on-one supportive counseling, while other patients might benefit from structured group formats. In addition, patients with rare medical conditions may feel isolated if placed in generic patient support groups, which may not adequately understand or relate to their health journey. When medical patients are offered a choice of social support options, such as peer mentoring, group sessions, family-inclusive approaches, or individualized therapy, they might also experience greater treatment engagement and adherence, which in turn should increase the efficiency and effectiveness of the intervention.

In addition, it is recommended that future research investigate team-based social support interventions in which both medical and mental health professionals provide patient care. Rather than relying on fragmented patient care, in which support facilitators work independently from other medical professionals, coordinated treatment among physicians, nurses, psychologists, and counselors may more effectively address holistic patient needs. Integrated care may be especially effective for patients with chronic health conditions, in which emotional well-being continually interacts with physical recovery and motivation.

In the present meta-analysis, only patient follow-up was associated with study effect size. No other patient and intervention characteristics (e.g., total intervention time, participant age, number of sessions, disease type) were associated with patient outcomes. As additional research is conducted over time, future reviews and meta-analyses may be able to determine which characteristics of interventions are the most effective.

Since interventions varied substantially across studies, it will be essential for future reviews to identify specific characteristics of the most effective interventions. For instance, social support interventions across the 14 studies in the current meta-analysis were often provided by peers or volunteers, but it seems possible that interventions involving family members could be more supportive and more effective than those delivered by previously unknown peers.

## 5. Conclusions

Chronic health conditions commonly result in patient social isolation and diminished emotional resilience, such that healthcare initiatives to improve patient social support may have substantial benefits [72]. The current meta-analysis examined the effectiveness of 14 non-randomized trials of social support interventions in improving the survival of medical patients, but averaging the small number of studies did not yield statistically significant results. Thus, the data available for this meta-analysis were limited and inconclusive, and future reviews will be needed to identify additional studies on the topic.

A notable finding was that the length of data follow-up significantly influenced the observed outcomes of the interventions. The longer the period of data collection, the more the survival benefit to medical patients receiving social support interventions became apparent. This finding implies that long-term observations may be necessary for the survival benefits of social support interventions to become apparent. Future research studies should track participants for at least two years and/or until at least 15% or more of participants have died to have sufficient cases for analyses. Overall, a future review with a larger pool of data and with studies exhibiting lower risk of research bias will be needed to establish firm conclusions.

## Figures and Tables

**Table 1:** Study descriptive information.

Study	Age	% Female	Condition	Months Followed	Effect Size	SE
Bottomley et al., 1996 [89]	53	77	Cancer	12	OR 0.21	1.59
Chambers et al., 2015 [90]	65	88	Cancer	5	OR 4.00	1.28
Dracup et al., 1984 [91]	57	10	CVD	6	OR 1.00	2.03
Fatti et al., 2012 [92]	35	69	HIV/AIDS	0	HR 1.54	0.06
Franke et al., 2013 [93]	37	62	HIV/AIDS	12	OR 1.77	0.36
Friedman et al., 1986 [94]	53	10	CVD	54	OR 2.45	0.33
Friend et al., 1986 [95]	49	43	Renal disease	120	HR 5.00	0.28
Gordon et al., 1980 [96]	52	68	Cancer	6	OR 0.64	0.42
Hickey et al., 2015 [97]	40	64	HIV/AIDS	22	OR 0.52	0.64
Hildingh et al., 2004 [98]	67	64	CVD	39	OR 2.42	0.78
Morgenstern et al., 1984 [99]	47	100	Cancer	26	HR 0.83	0.39
Rosendahl et al., 2013 [100]	66	22	CVD	22	OR 0.40	0.60
Shrock et al., 1999 [101]	65	42	Cancer	60	OR 3.16	0.56
Summers et al., 2000 [102]	37	100	HIV/AIDS	57	HR 3.05	0.44

Note: OR = odds ratio. HR = hazard ratio. SE = standard error.

**Table 2:** Evaluations of research risk of bias across ROBINS-I V2 domains.

Study	Confounding	Classification	Selection	Missing Data	Outcome Measure	Result Reporting	Overall Risk
Bottomley et al., 1996 [89]	Critical	Low	Low	Critical	Low	Moderate	Critical
Chambers et al., 2015 [90]	Serious	Low	Low	Critical	Low	Moderate	Serious
Dracup et al., 1984 [91]	Serious	Low	Low	Low	Low	Low	Low
Fatti et al., 2012 [92]	Moderate	Low	Low	Serious	Low	Low	Serious
Franke et al., 2013 [93]	Moderate	Low	Low	Low	Low	Low	Moderate
Friedman et al., 1986 [94]	Low	Low	Low	Serious	Low	Low	Serious
Friend et al., 1986 [95]	Moderate	Moderate	Low	Low	Low	Low	Moderate
Gordon et al., 1980 [96]	Critical	Low	Low	Critical	Low	Serious	Critical
Hickey et al., 2015 [97]	Moderate	Low	Low	Low	Low	Moderate	Moderate
Hildingh & Fridlund, 2004 [98]	Serious	Critical	Low	Critical	Low	Moderate	Critical
Morgenstern et al., 1984 [99]	Critical	Moderate	Serious	Critical	Low	Low	Critical
Rosendahl et al., 2013 [100]	Serious	Low	Low	Serious	Low	Low	Serious
Shrock et al., 1999 [101]	Serious	Low	Low	Low	Low	Low	Low
Summers et al., 2000 [102]	Serious	Critical	Moderate	Serious	Low	Low	Critical

## Data Availability

No new data were created or analyzed in this study. Data sharing is not applicable to this article.

## Abbreviations

The following abbreviations are used in this manuscript:

NRCT	Non-randomized control trials
RCT	Randomized controlled trials
CHP	Coronary heart disease
PICO	Population, intervention, comparison, outcome
OR	Odds ratio
HR	Hazard ratio
CVD	Cardiovascular disease
ROBINS-I	Risk of Bias in Non-randomized Studies—of Interventions

## References

[1] Baumeister R.F., Leary M.R. (1995). The Need to Belong: Desire for Interpersonal Attachments as a Fundamental Human Motivation. Psychol. Bull..

[2] Cacioppo S., Grippo A.J., London S., Goossens L., Cacioppo J.T. (2015). Loneliness: Clinical Import and Interventions. Perspect. Psychol. Sci..

[3] Umberson D., Karas Montez J. (2010). Social Relationships and Health: A Flashpoint for Health Policy. J. Health Soc. Behav..

[4] Drageset J., Haugan G., Eriksson M. (2021). Social Support. Health Promotion in Health Care—Vital Theories and Research.

[5] Hawkley L.C., Cacioppo J.T. (2010). Loneliness Matters: A Theoretical and Empirical Review of Consequences and Mechanisms. Ann. Behav. Med..

[6] Feeney B.C., Collins N.L. (2015). A New Look at Social Support: A Theoretical Perspective on Thriving Through Relationships. Pers. Soc. Psychol. Rev..

[7] Holt-Lunstad J. (2024). Social Connection as a Critical Factor for Mental and Physical Health: Evidence, Trends, Challenges, and Future Implications. World Psychiatry.

[8] Holt-Lunstad J., Smith T.B., Layton J.B. (2010). Social Relationships and Mortality Risk: A Meta-Analytic Review. PLoS Med..

[9] Vila J. (2021). Social Support and Longevity: Meta-Analysis-Based Evidence and Psychobiological Mechanisms. Front. Psychol..

[10] Murthy V. (2023). Our Epidemic of Loneliness and Isolation: The U.S. Surgeon General’s Advisory on the Healing Effects of Social Connection and Community.

[11] House J.S., Landis K.R., Umberson D. (1988). Social Relationships and Health. Science.

[12] Meluch A.L. (2018). Above and beyond: An Exploratory Study of Breast Cancer Patient Accounts of Healthcare Provider Information-Giving Practices and Informational Support. Qual. Res. Med. Healthc..

[13] Thoits P.A. (1995). Stress, Coping, and Social Support Processes: Where Are We? What Next?. J. Health Soc. Behav..

[14] Cutrona C.E., Russell D.W. (1990). Stress and Social Support—In Search of Optimal Matching. J. Soc. Clin. Psychol..

[15] Uchino B.N. (2004). Social Support and Physical Health: Understanding the Health Consequences of Relationships.

[16] Feinstein A.R., Horwitz R.I. (1997). Problems in the “Evidence” of “Evidence-Based Medicine”. Am. J. Med..

[17] Ware J.H., Hamel M.B. (2011). Pragmatic Trials—Guides to Better Patient Care?. N. Engl. J. Med..

[18] Glasgow R.E., Nutting P.A., King D.K., Nelson C.C., Cutter G., Gaglio B., Rahm A.K., Whitesides H., Amthauer H. (2004). A Practical Randomized Trial to Improve Diabetes Care. J. Gen. Intern. Med..

[19] Green L.W., Glasgow R.E. (2006). Evaluating the Relevance, Generalization, and Applicability of Research: Issues in External Validation and Translation Methodology. Eval. Health Prof..

[20] Victora C.G., Habicht J.-P., Bryce J. (2004). Evidence-Based Public Health: Moving Beyond Randomized Trials. Am. J. Public. Health.

[21] Sallis J.F., Glanz K. (2009). Physical Activity and Food Environments: Solutions to the Obesity Epidemic. Milbank Q..

[22] Barnes M.D., Hanson C.L., Novilla L.B., Magnusson B.M., Crandall A.C., Bradford G. (2020). Family-Centered Health Promotion: Perspectives for Engaging Families and Achieving Better Health Outcomes. INQUIRY.

[23] Ho Y.-C.L., Mahirah D., Ho C.Z.-H., Thumboo J. (2022). The Role of the Family in Health Promotion: A Scoping Review of Models and Mechanisms. Health Promot. Int..

[24] Michaelson V., Pilato K.A., Davison C.M. (2021). Family as a Health Promotion Setting: A Scoping Review of Conceptual Models of the Health-Promoting Family. PLoS ONE.

[25] Torres-Soto N.Y., Corral-Verdugo V., Corral-Frías N.S. (2022). The Relationship between Self-Care, Positive Family Environment, and Human Wellbeing. Wellbeing Space Soc..

[26] Meluch A.L. (2018). Narrating Patienthood: Engaging Diverse Voices on Health, Communication, and the Patient Experience.

[27] Southwick S.M., Bonanno G.A., Masten A.S., Panter-Brick C., Yehuda R. (2014). Resilience Definitions, Theory, and Challenges: Interdisciplinary Perspectives. Eur. J. Psychotraumatol..

[28] Boehm J.K. (2021). Positive Psychological Well-being and Cardiovascular Disease: Exploring Mechanistic and Developmental Pathways. Soc. Personal. Psych..

[29] Diener E., Wirtz D., Tov W., Kim-Prieto C., Choi D., Oishi S., Biswas-Diener R. (2010). New Well-Being Measures: Short Scales to Assess Flourishing and Positive and Negative Feelings. Soc. Indic. Res..

[30] Rashidi A., Kaistha P., Whitehead L., Robinson S. (2020). Factors That Influence Adherence to Treatment Plans amongst People Living with Cardiovascular Disease: A Review of Published Qualitative Research Studies. Int. J. Nurs. Stud..

[31] Rutter M. (1987). Psychosocial Resilience and Protective Mechanisms. Am. J. Orthopsychiatry.

[32] Wills T.A., Shinar O., Cohen S., Underwood L.G., Gottlieb B.H. (2000). Measuring Perceived and Received Social Support. Social Support Measurement and Intervention.

[33] Stanisławski K. (2019). The Coping Circumplex Model: An Integrative Model of the Structure of Coping With Stress. Front. Psychol..

[34] Yang R., Lu Z., Gu X., Dai B. (2021). The Effect of an Information Support Program on Self-Efficacy of Prostate Cancer Patients during Hormonal Therapy. Asia-Pac. J. Oncol. Nurs..

[35] Ruiz-Rodríguez I., Hombrados-Mendieta I., Melguizo-Garín A., Martos-Méndez M.J. (2022). The Importance of Social Support, Optimism and Resilience on the Quality of Life of Cancer Patients. Front. Psychol..

[36] Wilcox B.L., Vernberg E.M., Sarason I.G., Sarason B.R. (1985). Conceptual and Theoretical Dilemmas Facing Social Support Research. Social Support: Theory, Research and Applications.

[37] Evers A.W.M., Kraaimaat F.W., Geenen R., Jacobs J.W.G., Bijlsma J.W.J. (2003). Pain Coping and Social Support as Predictors of Long-Term Functional Disability and Pain in Early Rheumatoid Arthritis. Behav. Res. Ther..

[38] Cohen S. (1997). Social Ties and Susceptibility to the Common Cold. JAMA.

[39] Schultz B.E., Corbett C.F., Hughes R.G. (2022). Instrumental Support: A Conceptual Analysis. Nurs. Forum.

[40] Uchino B.N., Cawthon R.M., Smith T.W., Light K.C., McKenzie J., Carlisle M., Gunn H., Birmingham W., Bowen K. (2012). Social Relationships and Health: Is Feeling Positive, Negative, or Both (Ambivalent) about Your Social Ties Related to Telomeres?. Health Psychol..

[41] McEwen B.S., Schulkin J. (2004). Protective and Damaging Effects of the Mediators of Stress and Adaptation: Allostasis and Allostatic Load. Allostasis, Homeostasis, and the Costs of Physiological Adaptation.

[42] Seeman T.E., Singer B.H., Ryff C.D., Dienberg Love G., Levy-Storms L. (2002). Social Relationships, Gender, and Allostatic Load Across Two Age Cohorts. Psychosom. Med..

[43] Holtzman S., Newth S., Delongis A. (2004). The Role of Social Support in Coping with Daily Pain among Patients with Rheumatoid Arthritis. J. Health Psychol..

[44] Eisenberger N.I., Taylor S.E., Gable S.L., Hilmert C.J., Lieberman M.D. (2007). Neural Pathways Link Social Support to Attenuated Neuroendocrine Stress Responses. NeuroImage.

[45] Heinrichs M., Baumgartner T., Kirschbaum C., Ehlert U. (2003). Social Support and Oxytocin Interact to Suppress Cortisol and Subjective Responses to Psychosocial Stress. Biol. Psychiatry.

[46] Kreuter M.W., Thompson T., McQueen A., Garg R. (2021). Addressing Social Needs in Health Care Settings: Evidence, Challenges, and Opportunities for Public Health. Annu. Rev. Public. Health.

[47] Stewart M.J., Tilden V.P. (1995). The Contributions of Nursing Science to Social Support. Int. J. Nurs. Stud..

[48] Dorflinger L., Kerns R.D., Auerbach S.M. (2013). Providers’ Roles in Enhancing Patients’ Adherence to Pain Self Management. Behav. Med. Pract. Policy Res..

[49] Fonagy P., Allison E. (2014). The Role of Mentalizing and Epistemic Trust in the Therapeutic Relationship. Psychotherapy.

[50] Moudatsou M., Stavropoulou A., Philalithis A., Koukouli S. (2020). The Role of Empathy in Health and Social Care Professionals. Healthcare.

[51] Hojat M., Louis D.Z., Maxwell K., Markham F., Wender R., Gonnella J.S. (2010). Patient Perceptions of Physician Empathy, Satisfaction with Physician, Interpersonal Trust, and Compliance. Int. J. Med. Educ..

[52] Kwekkeboom K.L., Gretarsdottir E. (2006). Systematic Review of Relaxation Interventions for Pain. J. Nurs. Scholarsh..

[53] Smith T.B., Workman C., Andrews C., Barton B., Cook M., Layton R., Morrey A., Petersen D., Holt-Lunstad J. (2021). Effects of Psychosocial Support Interventions on Survival in Inpatient and Outpatient Healthcare Settings: A Meta-Analysis of 106 Randomized Controlled Trials. PLoS Med..

[54] Bhattad P.B., Pacifico L. (2022). Empowering Patients: Promoting Patient Education and Health Literacy. Cureus.

[55] Arsenault-Lapierre G., Henein M., Gaid D., Le Berre M., Gore G., Vedel I. (2021). Hospital-at-Home Interventions vs In-Hospital Stay for Patients With Chronic Disease Who Present to the Emergency Department: A Systematic Review and Meta-Analysis. JAMA Netw. Open.

[56] Bulto L.N. (2024). The Role of Nurse-led Telehealth Interventions in Bridging Healthcare Gaps and Expanding Access. Nurs. Open.

[57] Krumholz H.M. (2013). Post-Hospital Syndrome—An Acquired, Transient Condition of Generalized Risk. N. Engl. J. Med..

[58] King J., O’Neill B., Ramsay P., Linden M.A., Darweish Medniuk A., Outtrim J., Blackwood B. (2019). Identifying Patients’ Support Needs Following Critical Illness: A Scoping Review of the Qualitative Literature. Crit Care.

[59] Thomas T.C., Colburn T.A., Korp K., Khodadad A., Lifshitz J., Kobeissy F.H. (2015). Translational Considerations for Behavioral Impairment and Rehabilitation Strategies after Diffuse Traumatic Brain Injury. Brain Neurotrauma.

[60] Albus C., Theissen P., Hellmich M., Griebenow R., Wilhelm B., Aslim D., Schicha H., Köhle K. (2009). Long-Term Effects of a Multimodal Behavioral Intervention on Myocardial Perfusion—A Randomized Controlled Trial. Int.J. Behav. Med..

[61] Classen C., Butler L.D., Koopman C., Miller E., DiMiceli S., Giese-Davis J., Fobair P., Carlson R.W., Kraemer H.C., Spiegel D. (2001). Supportive-Expressive Group Therapy and Distress in Patients With Metastatic Breast Cancer: A Randomized Clinical Intervention Trial. Arch. Gen. Psychiatry.

[62] Ehde D.M., Dillworth T.M., Turner J.A. (2014). Cognitive-Behavioral Therapy for Individuals with Chronic Pain: Efficacy, Innovations, and Directions for Research. Am. Psychol..

[63] Goodkin K., Blaney N.T., Feaster D.J., Baldewicz T., Burkhalter J.E., Leeds B. (1999). A Randomized Controlled Clinical Trial of a Bereavement Support Group Intervention in Human Immunodeficiency Virus Type 1–Seropositive and –Seronegative Homosexual Men. Arch. Gen. Psychiatry.

[64] Grossman P., Kappos L., Gensicke H., D’Souza M., Mohr D.C., Penner I.K., Steiner C. (2010). MS Quality of Life, Depression, and Fatigue Improve after Mindfulness Training: A Randomized Trial. Neurology.

[65] Kissane D.W., Grabsch B., Clarke D.M., Smith G.C., Love A.W., Bloch S., Snyder R.D., Li Y. (2007). Supportive-expressive Group Therapy for Women with Metastatic Breast Cancer: Survival and Psychosocial Outcome from a Randomized Controlled Trial. Psycho-Oncology.

[66] Spiegel D., Giese-Davis J. (2003). Depression and Cancer: Mechanisms and Disease Progression. Biol. Psychiatry.

[67] Foley L., Larkin J., Lombard-Vance R., Murphy A.W., Hynes L., Galvin E., Molloy G.J. (2021). Prevalence and Predictors of Medication Non-Adherence among People Living with Multimorbidity: A Systematic Review and Meta-Analysis. BMJ Open.

[68] Levine D.M., Ouchi K., Blanchfield B., Saenz A., Burke K., Paz M., Diamond K., Pu C.T., Schnipper J.L. (2020). Hospital-Level Care at Home for Acutely Ill Adults: A Randomized Controlled Trial. Ann. Intern. Med..

[69] Megido I., Sela Y., Grinberg K. (2023). Cost Effectiveness of Home Care versus Hospital Care: A Retrospective Analysis. Cost. Eff. Resour. Alloc..

[70] Kanagala S.G., Gupta V., Kumawat S., Anamika F., McGillen B., Jain R. (2023). Hospital at Home: Emergence of a High-Value Model of Care Delivery. Egypt. J. Intern. Med..

[71] Mezey M., Fulmer T., McCorkle R., Strumpf N.E., Nuamah I.F., Adler D.C., Cooley M.E., Jepson C., Lusk E.J., Torosian M. (2000). ADVANCING GERIATRIC NURSING PRACTICE; A Specialized Home Care Intervention Improves Survival Among Older Post-Surgical Cancer Patients. J. Am. Geriatr. Soc..

[72] Yu B., Steptoe A., Chen L.-J., Chen Y.-H., Lin C.-H., Ku P.-W. (2020). Social Isolation, Loneliness, and All-Cause Mortality in Patients With Cardiovascular Disease: A 10-Year Follow-up Study. Psychosom. Med..

[73] Schünemann H.J., Tugwell P., Reeves B.C., Akl E.A., Santesso N., Spencer F.A., Shea B., Wells G., Helfand M. (2013). Non-randomized Studies as a Source of Complementary, Sequential or Replacement Evidence for Randomized Controlled Trials in Systematic Reviews on the Effects of Interventions. Res. Synth. Methods.

[74] Deeks J., Dinnes J., D’Amico R., Sowden A., Sakarovitch C., Song F., Petticrew M., Altman D. (2003). Evaluating Non-Randomised Intervention Studies. Health Technol. Assess..

[75] Harris A.D., McGregor J.C., Perencevich E.N., Furuno J.P., Zhu J., Peterson D.E., Finkelstein J. (2006). The Use and Interpretation of Quasi-Experimental Studies in Medical Informatics. J. Am. Med. Inform. Assoc..

[76] Shadish W.R., Cook T.D., Campbell D.T. (2002). Experimental and Quasi-Experimental Designs for Generalized Causal Inference.

[77] West S.G., Duan N., Pequegnat W., Gaist P., Des Jarlais D.C., Holtgrave D., Szapocznik J., Fishbein M., Rapkin B., Clatts M. (2008). Alternatives to the Randomized Controlled Trial. Am. J. Public. Health.

[78] Benson K., Hartz A.J. (2000). A Comparison of Observational Studies and Randomized, Controlled Trials. N. Engl. J. Med..

[79] Guillaume B., Hua X., Thompson P.M., Waldorp L., Nichols T.E. (2014). Fast and Accurate Modelling of Longitudinal and Repeated Measures Neuroimaging Data. NeuroImage.

[80] Ioannidis J.P.A. (2001). Comparison of Evidence of Treatment Effects in Randomized and Nonrandomized Studies. JAMA.

[81] Pawson R., Tilley N. (1997). An Introduction to Scientific Realist Evaluation. Evaluation for the 21st Century: A Handbook.

[82] Reeves B.C., Deeks J.J., Higgins J.P., Shea B., Tugwell P., Wells G.A., Higgins J.P.T., Thomas J., Chandler J., Cumpston M., Li T., Page M.J., Welch V.A., on behalf of the Cochrane Non-Randomized Studies of Interventions Methods Group (2019). Including Non-Randomized Studies on Intervention Effects. Cochrane Handbook for Systematic Reviews of Interventions.

[83] Efthimiou O., Mavridis D., Debray T.P.A., Samara M., Belger M., Siontis G.C.M., Leucht S., Salanti G., on behalf of GetReal Work Package 4 (2017). Combining Randomized and Non-randomized Evidence in Network Meta-analysis. Stat. Med..

[84] MacPherson M., Rourke S. (2024). The Power of Rapid Reviews for Bridging the Knowledge-to-Action Gap in Evidence-Based Virtual Health Care. J. Med. Internet Res..

[85] Devane D., Hamel C., Gartlehner G., Nussbaumer-Streit B., Griebler U., Affengruber L., Saif-Ur-Rahman K.M., Garritty C. (2024). Key Concepts in Rapid Reviews: An Overview. J. Clin. Epidemiol..

[86] Chalmers I., Glasziou P. (2009). Avoidable Waste in the Production and Reporting of Research Evidence. Lancet.

[87] Ewald H., Klerings I., Wagner G., Heise T.L., Dobrescu A.I., Armijo-Olivo S., Stratil J.M., Lhachimi S.K., Mittermayr T., Gartlehner G. (2020). Abbreviated and comprehensive literature searches led to identical or very similar effect estimates: A meta-epidemiological study. J. Clin. Epidemiol..

[88] Wilson D.B. (2023). Practical Meta-Analysis Effect Size Calculator (Version 2023.11.27). https://www.campbellcollaboration.org/calculator/.

[89] Bottomley A., Hunton S., Roberts G., Jones L., Bradley C. (1996). A pilot study of cognitive behavioral therapy and social support group interventions with newly diagnosed cancer patients. J. Psychosoc. Oncol..

[90] Chambers S.K., Morris B.A., Clutton S., Foley E., Giles L., Schofield P., O’Connell D., Dunn J. (2015). Psychological wellness and health-related stigma: A pilot study of an acceptance-focused cognitive behavioural intervention for people with lung cancer. Eur. J. Cancer Care.

[91] Dracup K., Meleis A.I., Clark S., Clyburn A., Shields L., Staley M. (1984). Group counseling in cardiac rehabilitation: Effect on patient compliance. Patient Educ. Couns..

[92] Fatti G., Meintjes G., Shea J., Eley B., Grimwood A. (2012). Improved survival and antiretroviral treatment outcomes in adults receiving community-based adherence support: 5-year results from a multicentre cohort study in South Africa. J. Acquir. Immune Defic. Syndr..

[93] Franke M.F., Kaigamba F., Socci A.R., Hakizamungu M., Patel A., Bagiruwigize E., Niyigena P., Walker K.D.C., Epino H., Binagwaho A. (2013). Improved retention associated with community-based accompaniment for antiretroviral therapy delivery in rural Rwanda. Clin. Infect. Dis..

[94] Friedman M., Thoresen C.E., Gill J.J., Ulmer D., Powell L.H., Price V.A., Brown B., Thompson L., Rabin D.D., Breall D.D. (1986). Alteration of type A behavior and its effect on cardiac recurrences in postmyocardial infarction patients: Summary results of the recurrent coronary prevention project. Am. Heart J..

[95] Friend R., Singletary Y., Mendell N.R., Nurse H. (1986). Group participation and survival among patients with end-stage renal disease. Am. J. Public. Health.

[96] Gordon W.A., Freidenbergs I., Diller L., Hibbard M., Wolf C., Levine L., Lipkins R., Ezrachi O., Lucido D. (1980). Efficacy of psychosocial intervention with cancer patients. J. Consult. Clin. Psychol..

[97] Hickey M.D., Salmen C.R., Omollo D., Mattah B., Fiorella K.J., Geng E.H., Bacchetti P., Blat C., Ouma G.B., Zoughbie D. (2015). Pulling the network together: Quasi-experimental trial of a patient-defined support network intervention for promoting engagement in HIV care and medication adherence on Mfangano Island, Kenya. J. Acquir. Immune Defic. Syndr..

[98] Hildingh C., Fridlund B. (2004). A 3-year follow-up of participation in peer support groups after a cardiac event. Eur. J. Cardiovasc. Nurs..

[99] Morgenstern H., Gellert G.A., Walter S.D., Ostfeld A.M., Siegel B.S. (1984). The impact of a psychosocial support program on survival with breast cancer: The importance of selection bias in program evaluation. J. Chron. Dis..

[100] Rosendahl J., Tigges-Limmer K., Gummert J., Dziewas R., Albes J.M., Strauss B. (2013). Bypass surgery with psychological and spiritual support (the BY.PASS Study): Results of a pragmatic trial based on patients’ preference. Psychother. Psychosom..

[101] Shrock D., Palmer R.F., Taylor B. (1999). Effects of a psychosocial intervention on survival among patients with stage I breast and prostate cancer: A matched case-control study. Altern. Ther. Health Med..

[102] Summers J., Robinson R., Capps L., Zisook S., Atkinson J.H., McCutchan E., Mccutchan J.A., Deutsch R., Patterson T., Grant I. (2000). The influence of HIV-related support groups on survival in women who lived with HIV: A pilot study. Psychosomatics.

[103] ROBINS-I V2 Development Group (2025). Risk of Bias Tools—ROBINS-I V2 Tool. https://www.riskofbias.info/welcome/robins-i-v2.

[104] Stromberg A. (2003). Nurse-Led Heart Failure Clinics Improve Survival and Self-Care Behaviour in Patients with Heart failure: Results from a Prospective, Randomised Trial. Eur. Heart J..

[105] Ruan T., Xu M., Zhu L., Ding Y. (2023). Nurse-Coordinated Home-Based Cardiac Rehabilitation for Patients with Heart Failure: A Scoping Review. Int. J. Nurs. Sci..

[106] Kazdin A.E. (2023). Drawing Causal Inferences from Randomized Controlled Trials in Psychotherapy Research. Psychother. Res..

[107] Antoni M.H., Wimberly S.R., Lechner S.C., Kazi A., Sifre T., Urcuyo K.R., Phillips K., Smith R.G., Petronis V.M., Guellati S. (2006). Reduction of Cancer-Specific Thought Intrusions and Anxiety Symptoms With a Stress Management Intervention Among Women Undergoing Treatment for Breast Cancer. AJP.

[108] DiMatteo M.R., Lepper H.S., Croghan T.W. (2000). Depression Is a Risk Factor for Noncompliance With Medical Treatment: Meta-Analysis of the Effects of Anxiety and Depression on Patient Adherence. Arch. Intern. Med..

[109] Grenard J.L., Munjas B.A., Adams J.L., Suttorp M., Maglione M., McGlynn E.A., Gellad W.F. (2011). Depression and Medication Adherence in the Treatment of Chronic Diseases in the United States: A Meta-Analysis. J. Gen. Intern. Med..

[110] Ludolph P., Kunzler A.M., Stoffers-Winterling J., Helmreich I., Lieb K. (2019). Interventions to Promote Resilience in Cancer Patients. Dtsch. Ärzteblatt Int..

[111] Andersen B.L., Yang H., Farrar W.B., Golden-Kreutz D.M., Emery C.F., Thornton L.M., Young D.C., Carson W.E. (2008). Psychologic Intervention Improves Survival for Breast Cancer Patients: A Randomized Clinical Trial. Cancer.

[112] Holtmaat K., Van Der Spek N., Lissenberg-Witte B., Breitbart W., Cuijpers P., Verdonck-de Leeuw I. (2020). Long-term Efficacy of Meaning-centered Group Psychotherapy for Cancer Survivors: 2-Year Follow-up Results of a Randomized Controlled Trial. Psycho-Oncology.

